# Taking sides or bridging worlds? Managerial responses to conflicts and tensions between the core operations and the administration in healthcare

**DOI:** 10.1186/s12913-025-13659-9

**Published:** 2025-11-08

**Authors:** Ingrid Svensson

**Affiliations:** LIME, Karolinska Institutet, Solna, Sweden

**Keywords:** Healthcare managers, Conflicts, Power, Skills, Tensions, Public professionals, Core operations, Administration

## Abstract

**Background:**

Swedish public healthcare is increasingly characterised by a growing administrative layer and complex governance structures. While administrative personnel have grown in numbers, their coordination and integration with core clinical operations appears to remain underdeveloped, leading to tensions, conflicts and perceived power vacuums. Despite the growing complexity, little research has focused on how healthcare managers, including those outside the clinical line, navigate these tensions. This study addresses this gap by exploring how managers understand and respond to conflicts between administrative and clinical domains, and how these dynamics affect their ability to act as public professionals with ‘holistic viewpoints’ within public healthcare.

**Methods:**

The study employed qualitative methods, including semi-structured interviews with 24 healthcare managers across three Swedish regions. Participants represented both clinical line organisations and administrative units across hierarchical levels. Thematic reflexive analysis was used to identify patterns in how managers perceive and navigate tensions and conflicts, with a focus on power, conflict resolution, and perception of where one’s loyalties lie.

**Results:**

The results show how managers often distanced themselves from responsibility for resolving conflicts and managing tensions, delegating integrative tasks to roles with limited mandates. First-line clinical managers aligned with their staff, reinforcing divisions between administration and core operations, and were shown to have much power in practice, yet did not recognise this. Administrative managers lacked legitimacy and formal authority, something which complicated collaboration. Education was proposed as a solution to tensions and conflicts, but often in vague terms. Power was perceived as always residing elsewhere, either with top management, politicians or core operative personnel. While some managers experienced a shift in understanding when moving up the hierarchy, this did not consistently translate into integrative practices. Overall, the findings reveal a gap between the recognition of tensions and conflicts and the capacity or willingness to address them constructively in line with how current research frames ‘public professionals’. Managers’ actions often reinforced rather than bridged divides, reflecting a lack of holistic perspective amongst healthcare managers.

**Conclusions:**

Healthcare managers struggle to act as public professionals in a fragmented system marked by conflicting logics and unclear mandates. Rather than bridging divides, many reinforce them through alignment with their groups or delegation. Addressing these challenges requires rethinking managerial roles and fostering strategic, integrative leadership across organisational boundaries for healthcare organisations.

**Supplementary information:**

The online version contains supplementary material available at 10.1186/s12913-025-13659-9.

## Introduction

A healthcare manager recounted a situation in which representatives from the administration posted on social media about a recent initiative they considered a success. However, from the perspective of staff in the core operations, the same initiative was seen as a failure. The manager reflected on the tension this created and on the challenges of navigating such dynamics, particularly given the criticism directed at the administration by core staff. *They [the administration] are also a group of employees who have succeeded in various things. But in the general debate … there is no understanding of that at all/ … ./So, the administrative staff also need help and support …. It is a difficult dynamic, where I, in my role, must constantly help, support, and raise these kinds of issues. (Top manager, Hospital).* This quote illustrates how ongoing tensions and conflicting perspectives between the administration and core operations affect healthcare managers, prompting reflection on their roles and responsibilities in mediating between these groups.

Public healthcare managers today operate at the intersection of multiple governance modes, including traditional public administration, new public management, and new public governance [1]. These intersecting modes of governance place complex demands on public managers, who ought to navigate not only between professional and managerial expectations but also across fragmented systems of accountability, legitimacy, and coordination. Here, public managers are expected to be ‘public professionals’ who bridge worlds [1] and employ a holistic viewpoint [2]. At the same time, recent reforms in healthcare organisations have tended to consolidate the authority of the core operations rather than challenging it (cf. [3], something that contributes to tensions and conflicts between those actors that belong to the increasing so-called ‘administration’ in healthcare, the core operations and their managers.

In recent years, ‘the administration’, comprising highly skilled administrative personnel [4] within public organisations, including healthcare, has expanded significantly in Sweden. In this paper, those managers who are working in what Glouberman and Mintzberg [5] would label ‘the technostructure’ and ‘support staff’, are collectively referred to as ‘the administration’ to distinguish them organisationally from the line organisation, which includes the core operations. Yet, paradoxically, as this administrative layer has grown, there are increasing signs that no one is steering it. With increased pressure on collaboration and new ‘connective ideals’ being prominent in public organisations [6] there needs to be someone to manages these connections, for example between ‘the administration’ and the core operations, yet, here, managers are perceived as absent, and individual officials themselves must define their areas of responsibility, something which is difficult in practice [4]. Research has shown how intensification of the ‘management’ logic has occurred in public healthcare organisations without a significant increase in the number of managers employed as a ‘distinct occupation’; hospitals that have transitioned to ‘complete’ organisations shows that they become both ‘managed’ and under-managed at the same time [7]. Likewise, in Sweden, it has been argued that there is a lack of steering of the administration [4], resulting in a ‘control vacuum’ for the growing administration [8].

In parallel with this control vacuum, tensions between administrative structures and the line organisations have become more visible in healthcare organisations [9]. Sometimes resulting in conflicts. Tensions are here defined as emotional strains that affect behaviours. The concept of tensions is “explicitly addresses the relational aspects of manoeuvring conflicts and challenges” [10, p. 2]. In line with Rossi and Tuurna [2], conflict in this paper is defined as an individual’s experience and understanding of a given situation or phenomenon, which is different from that of others involved. Tensions and conflicts are, for example, manifested in practice between clinicians and non-clinicians in terms of perceptions of value and power [11, 12].

Despite attention to an increase in tensions and conflicts between core operations and the administration (cf. [9]), little research has examined the role of managers in navigating these. While research has been attentive to the fact that so-called highly administrative personnel is increasing in public organisations and why this is so [4], that clinicians and non-clinicians are distant in practice [12] and that it is difficult for individuals administrative officials to find their way to perform their work in this new situation resulting in tensions between occupational groups [9], less research has explored the role of managers in this situation and how their behaviours and perceptions bridge organisational divides or contribute to their separateness.

This paper addresses this gap by focusing on the role of healthcare managers in tensions and conflicts related to the growing administration. It does so by shifting the analytical lens beyond the dominant focus on healthcare managers in the line organisation, being understood as hybrids who are to balance managerial and professional logics (e.g. [13–15]). While this binary framing has been useful to account for what it is like to be a manager in healthcare, typically when having a clinical background, it risks oversimplifying the structural and systemic complexity in which public healthcare managers operate (cf. [16]) and does not include those healthcare managers who are not working as former (or current) clinicians and who work in ‘the administration’.

By exploring how Swedish public healthcare managers, both from ‘the administration’ and the line organisation, understand and navigate tensions and conflicts between ‘the administration’ and the core operations/line-organisation(s), this paper contributes to a nuanced understanding of the challenges facing healthcare managers in today’s public healthcare organisations. The reason to focus on tensions and conflicts is twofold. First, tensions between ‘the line organisation’ and ‘the administration’ have of late increased in Swedish healthcare (cf. [9]), but the role of managers in and for these tensions has not been investigated. Second, conflicts are a fundamental part of everyday organisational life, including the complex social systems in which public organisations, such as healthcare organisations, are embedded. It is increasingly acknowledged that public service providers need to balance between different values, for example, of legality, efficiency, effectiveness, and professionalisation, causing conflicts between actors within public organisations (cf. [2]). For healthcare managers to be able to be reflexive public professionals [1] and employ a ‘holistic viewpoint’, it is important to reveal how conflicts between organisational groups and units are being experienced and dealt with (cf. [2]).

To this end, the paper addresses the following research questions: How do healthcare managers, both those representing ‘the line organisations’ and those representing ‘the administration’ navigate tensions and conflicts between them, and how does this affect the possibility of acting like reflexive public professionals?

## Previous research

### Healthcare managers

Previous research on healthcare managers has typically focused on managers in the line organisation, i.e. managers for the core operations. The traditional view of these healthcare managers is that they tend to act as diplomats to ensure the organisation runs smoothly and that doctors’ work is facilitated in their core mission: direct patient care [17]. In public organisations, including healthcare, line managers have often emerged from the core operations and have traditionally had the task of defending the independence of welfare work against the control of management and administration [18]. Historically, these managers have acted as buffers or diplomats, defending professional autonomy against administrative control [17]. At the same time, their jurisdiction is less clearly defined than that of clinicians, and their authority is often questioned or bypassed [11]. Line managers are primarily perceived as colleagues and therefore not asked for advice on managerial issues [19], which leads to clinicians not perceiving their direct manager as an effective structural link between themselves and their organisation, and employees feel that their managers have little influence over their work situation [19].

In healthcare, managerial authority is typically secondary to professional status [3], and nurses have labelled their managerial work ‘mysterious work’ [20]. Physicians have typically been responsible for an obvious, legitimate area of tasks that is rarely disputed by other professional groups, and they tend to keep this focus as they become managers [19]. One strategy to engage physicians in organisational processes in the past has been to give them a lot of authority, without formal training in organisational issues [21]. Since many healthcare managers come from a health profession, they are not formally trained in organisational development or coordination work [22]. For example, physicians have been shown to lack knowledge of the system they are in [19]. Several studies have found that the strong medical profession tends to criticise increased management and use effective tactics to remain independent [23]. This aligns with Kirkpatrick et al. [3], who argue that despite decades of reform, the formal consolidation of managerial hierarchies (“the line”) has not displaced the informal dominance of elite professionals.

### Different professional groups in healthcare and their relations

Traditionally, in healthcare organisations, clinicians and particularly physicians have been highly autonomous, since they, through their professional associations, have been controlling their work [24]. However, of late, this control is challenged by other types of occupational groups, which has led to increased tensions between occupational groups in healthcare. Professional groups are constantly reconfiguring around new issues [15]. In healthcare, these groups are healthcare professionals such as physicians, nurses and other types of clinical personnel, but of late, also, increasingly, economists, managers, or policymakers [25]. Organisational rather than professional control is on the rise in public organisations of today [26], in healthcare for example, seen through intensified means from the administration to standardise the core operations’ work [27] and so-called organisational professionals, those that work with highly skilled administration, organisational coordination and development, in the public debate often grouped as ‘the administration’, have been argued to gain increased status and control [4]. Often, attempts are made to make different groups collaborate, but this is difficult in practice. Inclusion and communication between occupational groups may also sound good in theory, but in practice may entail continued distance. For example, in a study exploring the amendment of planning practices, increasing communication between clinicians and administrators meant: “getting them [other occupational group] onboard”/“making them understand” [our viewpoint] [11].

More, while for members of ‘the administration’, their organisational identification is commonly that of their organisations, traditional professions, such as physicians, identify with their profession or their close professional group [17]. Thus, for a healthcare manager, their personnel’s organisational identification cannot be taken for granted, especially when personnel are deeply committed to another work-related professional group. Physicians identify strongly with their profession and weakly with the organisation - or even see managers as an out-group with antithetical values [28].

### Perception of power in healthcare organisations

For public organisations, it has been argued that several ‘organszations’ or ‘sub-systems’ are being built simultaneously within the boundaries of a single organisation, such as the core business, HR department, financial organization, development organization. These entities develop their own ‘in-groups’ that become increasingly distant from the core operations. While production in the core operation is managed [29], no one seems to be guiding the administration and its relationship with the core operations. In healthcare, some managers may lack professional legitimacy and/or belong to a subsystem not regarded as valuable as other subsystems within healthcare [30]. This can affect their capacity to influence organisational processes, despite having a formal mandate. How the professional hierarchy is negotiated influence leadership practices [31], and the presence of, for example, conflicting logics is not necessarily problematic, but rather how these conflicting perspectives are managed [32].

The expansion of ‘the administration’ has been attributed to ‘external forces’ [4]. (External) accountability and ‘the law’ have been argued as reasons for the increase in highly skilled administrators by officials at first- and second-line levels [29] and both managers and clinicians in healthcare tend to believe that they are subject to external forces that they cannot influence, such as laws and regulations [12]. In healthcare organisations, previous research has shown how it has been common to assume that decisions have been made by managers when, in fact, other groups have been responsible. Martin et al. [12] showed the existence of mutually incompatible fantasies of ‘the Other’, by both clinicians and the administration. They also exemplified the differences between clinical and non-clinical leaders, referring to ‘a constant barrier’. It has also been shown how organisations tend to leave the task of coordination and establishing connective practices to individual ‘administrators’ who lack an organisational mandate to connect different organisations [6].

Research has shown that being a broker in healthcare is hard [22]. This is because practices of inclusion are understood differently at various levels and parts of the healthcare system [33]. According to Vik and Hansson [22], a manager can assume the role of promoting as well as inhibiting inclusion. Inclusive managers play a key role in healthcare organisations when being so-called brokers, i.e. actors providing a shared experience and transcending the system boundaries between different actors. However, to succeed with this, healthcare managers must have authority in all groups to which they belong and work.

### Healthcare professionals in an increasingly complex institutional context, and the skills needed in this situation

Hendrikx et al. [1], in their literature review on strategic renewal in public services, argue that successive waves of public management reform, spanning Traditional Public Administration, New Public Management, and New Public Governance, have produced layered and often conflicting role expectations for public professionals. Hendrikx et al. [1] identified four key skill domains needed for public professionals to be ‘reflexive’: collaborative, communicative, strategic, and enabling skills, which reflect the expanded scope of professional responsibility in contemporary public service delivery. In more detail, the following skills were identified by Hendrikx et al. [1]; collaborative skills (receptiveness to new ideas, empathy, patience, diplomacy and ability to understand and integrate diverse perspectives), communication and mediation skills (effective cross-sector communication, conflict resolution and negotiation, bridging different professional and organisational logics); strategic leadership skills (vision-setting and storytelling, designing collaborative structures, navigating performance metrics and institutional complexity) enabling skills (facilitating inclusive and productive environments and engaging political actors and securing support). These findings underscore the need to move beyond the classical hybridity model of healthcare managers, based on their ability to navigate managerial and professional logics, and instead recognise healthcare managers as institutional actors who mediate between policy, practice, and public value creation [1]. By framing healthcare managers as reflexive public professionals, the focus shifts from internal organisational tensions to their strategic role in shaping and delivering public value across organisational boundaries and various sub-systems. Rather than balancing professional and managerial logics, professionals are framed as actors expected to act as collaborative partners in networked governance, engaging in co-creation with stakeholders across institutional boundaries. They are simultaneously guardians, autonomous experts working within bureaucratic structures; service providers, focused on efficiency, performance, and customer orientation, and collaborative partners, engaged in co-creation and networked governance with citizens and other stakeholders. These roles are not mutually exclusive; professionals often need to juggle multiple, sometimes conflicting, expectations. Here, Eriksson and Andersson [16] show how public officials may use the new public management logic to address new public governance ideals of collaboration and value co-creation. Consequently, they need to be able to carefully reconcile conflicting expectations.

## Method

### Data collection

The context for the study was Swedish healthcare Regions. In Sweden, the Regions are the overarching authority for healthcare in the country, including acute care hospitals, psychiatric care, and primary healthcare. Semi-structured interviews were conducted with managers at various levels of organisational and administrative units, including hospitals and primary healthcare centres in three Regions. The managers interviewed were thus both employed in ‘the line’ and in other organisational units. The sampling was a combination of purposive and snowball, where the aim was to cover various parts of healthcare hierarchies and their sub-systems. The interviews were semi-structured and based on two main topics: the role as manager and the relation to current tensions and conflicts between the core operations/the line organisation and ‘the administration’ in healthcare. The interview guide can be found in Appendix A.

**Fig. 1:**
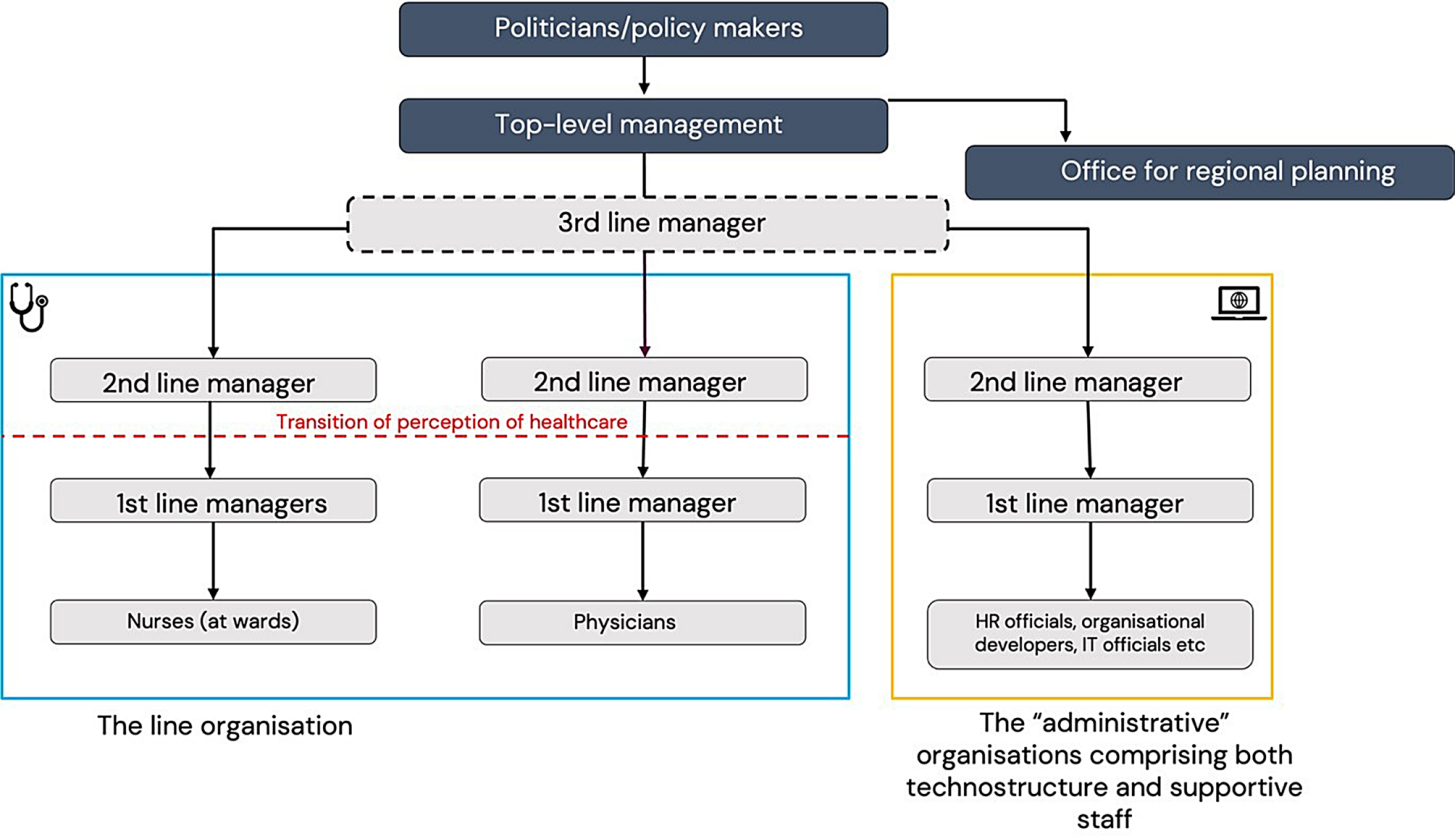
Illustrative, simplified image of hierarchical and organisational levels and units in Swedish Regions

Figure 1 provides a simplified, illustrative organisational chart of the organisations in which the managers worked. The illustration aims to show the difference between the line organisation and ‘the administration’. Notably, Fig. 1 is illustrative and a simplified model. For example, not only nurses and physicians worked in organisations included in the study, and the administrative organisations comprised different units and additional layers.

All managers, except two managers in the line organisation, were employed as full-time managers. Some of the first-line managers in the line organisation, however, conducted clinical work when they felt that there was a need for it. For an overview of interviewees, see Table 1.

**Table 1:** Overview of interviewees

Interviewees	Background	Region 1	Region 2	Region 3
First-line managers (Line organisation/core operations)	Former clinicians	7	2	
Second-Line managers (Line organisation/core operations)	Former clinicians	2	2	1
Director (Hospital, Region)	Former clinicians	2	2	
The equivalent of a first-line Manager Administrative organisation	Other educational background than a clinician (Engineer, management, finance and/or equivalent)	1		
The equivalent of a second-line Manager Administrative organisation	Other educational background than a clinician (Engineer, management, finance and/or equivalent)	2	3	

The interviews, which were all 60 minutes in length, were conducted either in person or online using Teams or Zoom and transcribed verbatim. The interviews focused on the interviewees’ perceptions and reasoning regarding the ongoing tensions between ‘the administration’ and the core operations, and how they respond to acknowledged conflicts between these occupational groups.

### Data analysis

The data analysis was inductive in that the themes were derived from the data and conducted using thematic reflexive analysis [34]. The data analysis was conducted in three overarching steps. First, the material was read through to get a sense of it with the broad question of: What is the role of managers in and for tensions and conflicts between the administration and the core operations? Thereafter, extracts that dealt with managers’ reasoning on tensions and conflicts between ‘the core operations’ and ‘the administration’ were elucidated.

The extracts were carefully read through and coded. Here, attention was given to the different types of managers interviewed and the different hierarchical levels of the organisation. From this initial coding process, sub-themes were created that were later categorised into three overarching themes that included all types of managers. Here, the analysis was close to the data, and the interviewee’s own words were used to define, for example, what was ‘deemed’ as ‘the administration’, which varied between interviewees (cf. [35]). The administration was a common word and concept mentioned in the interviews. However, the meaning of the concept varied. This was related to the perception that it was a unified ‘thing’ in which, at the organisational level, administrative units could be merged without further considerations. Second, the managers also varied in what they considered to be ‘the administration’, if it was included in the line or not, and where it ‘starts’/where are its boundaries. While some managers would define anything above first-line managers as part of ‘the administration’, others would define all roles outside the line ‘the administration’. First-line managers would argue that being for example in the top position at the hospital, despite being a doctor by training, “you *are* not a doctor once you have taken such a position”, you are part of the administration. However, while the first-line managers argued that the managers above them did not know anything about their reality and patient work, the highest manager in a large healthcare organisation takes pride in:

 “*Being bedside in my mind” … I think patient, patient, patient.* Other top managers would also argue that they simply did not understand the idea that the hospital is not run by professionals, and did not understand thamselves to be part of ‘a non professional’ administration “*I am a physician, and I run this hospital; thus, the profession is running the hospital*. In the findings, it is specified what meaning of ‘the administration’ is referred to on each occasion when deemed necessary.

 In addition to the managers’ interviews, data from a larger project addressing tensions between occupational groups in healthcare, and their implications, were reviewed. In this material, interviews with employees were also included. This material was not coded in detail like the interview material but was used to contextualise the initial findings. Data from one informant is included as a quote, for that purpose, in this paper. The rest of this data was read as a validation of the results. Some of that data is presented in Svensson and Andersson [9]. From the extracted data, three themes were captured. Within each themes, the analysis highlights the nuanced experiences of different types of managers across various hierarchical levels.

In the third and final step, the literature on healthcare managers and public management was revisited and related to the findings. Here, the guiding question was: How managers navigate the tensions and conflicts between the administration and the core operations and how this, in turn, affects their ability to act like reflexive public professionals [1].

## Findings

### Healthcare managers’ attempts at easing tensions and solving conflicts

#### Aiming to solve tensions through roles other than managers

The need for close collaboration between the administration and the line organisation, to avoid conflicts and tensions, was mentioned by the managers. However, a recurring means was to put the responsibility for this collaboration on other roles, rather than themselves as managers. The extract below illustrates a line of reasoning in which challenges in collaboration between the core operations and the administration are attributed to specific roles, particularly those associated with the expanding administrative sector

Third-line clinical manager: *[High] quality of the administrative service … can only be achieved if there is close cooperation between ‘the administrators’ and us and as well as responsiveness to what we need.*

Interviewer: *What was/is your role [in facilitating this collaboration and managing the situation]? What could you do?*

Third-line manager: *There are organisational developers … they fulfil a strategic function, as well as relieving the burden and getting operations going in projects and so on. We don’t have a lot of them here, but we have a few … [and] it is important to create personal relationships with them.*

In the same vein, physicians and nurses in the core operations described a reliance of organisational developers to solve organisational issues by their managers. One physician, who worked 20% with the development of care processes, describes how she is lonely in this role, and no one is there to help her navigate the organisational structures necessary to make change happen. She says that while her managers support her in one way, she cannot help with overarching organisational issues: *From my clinical manager … I get the hours that I need, and that is no problem … but … it is not like that is someone I can work with concerning implementation …*

What has happened, she describes, is the increase in administrative personnel, like ‘organisational developers’ who are to support organisational issues. However, *those [i.e. the organisational developers] who are to help … who are employed to work with development issues, they have even weaker mandates than I … They [the organisation] have not solved this issue …*

Another recurrent means to solve conflicts was to escalate conflicts to the top management. On occasions when the highest managers in the administration did not manage to solve issues with their ‘counterparts’ in the line organisation, they had to turn to members of top management, who governed both the administration and the line organisation. These were usually former clinicians, mainly physicians, and, although not seen as ‘real’ physicians, their professional status, together with their place in top management, usually made them able to solve conflicts at least in the short term and concerning specific issues.

#### Aiming to ease tensions through education

One conflicting issue that the managers needed to navigate was that of competence regarding the administration. There were notable differences between all types of managers in their opinions about who should work with ‘administrative’ issues in healthcare. Some managers in the line organisation alluded to the idea that people with medical education should be the ones working with ‘administration’, whereas others did not see it this way. One third-line manager in the line organisation said: *“We would never accept that in any other area [in healthcare], why do we accept it when it comes to fire safety and those issues … that [for example] nurses work with these things … when they have no education for it?” (Third-line manager, physician).* Other line managers had the direct opposite opinion: *“The best to work in finance in healthcare are former nurses or physicians!”.* Another manager, a physician by training and now responsible for several care organisations, said, concerning how she chooses who should be doing development work (as one example of an ‘administrative task’): *- [the] organisational developers have been chosen wisely, they are incredibly talented clinicians! They have a passion for development!* In this context, passion appears to be considered sufficient for engaging in development work, rather than formal education in the subject. Overall, while the clinical personnel are guided by formal rules regarding roles and responsibilities, the competencies required within the administration were not regulated, leading to tensions and conflicting views.

To solve the conflict and tensions currently present, one suggestion given was to educate both clinicians and ‘the administration’ about each other and each other’s roles: One healthcare director describes: *We are trying to build on the classic health profession training with additional skills … and we need to do the same in the other direction. So, if an engineer or an economist or whatever comes in, to increase confidence, you need to build up skills for their sake, linked to why we work in healthcare? I think there is a key … .*

The first-line managers interviewed explained that this is where the” real” work gets done. Summarising their perceptions, anything above the first line could be conceptualised as ‘the administration far away’. When/if moving from 1st line to 2nd line manager, physicians and nurses describe undergoing a “transition”. They start to see healthcare as a system and thereby appreciate the administrative staff more. This step was notably distinct, and the gap between the first- and second-line seemed large and as if between different worlds. Once having made this ‘transition’, these line organisation managers emphasised that the clinicians were the ones who needed to be educated, but that this was done less formally, here a top management administrative manager said: *I need to teach the physicians about the system*. Another one (top manager, former physician, line organisation) adds: *I take on the role as teacher in how society functions when I meet junior doctors.* This latter view seemed to be an ‘epiphany’ for those former clinicians who moved beyond first-line managers. First-line managers, on the other hand, were keener on educating ‘the administration’ about ‘the real world’.

### Conflicting loyalties

#### Not being a manager for the administration poses problems for managers in the line organisation

Line organisation managers described challenges in integrating administrative staff into their operations, as they were not the staff’s formal manager. A second-line manager in the line organisation says: *I cannot manage and distribute their [the administrative people’s] tasks*, *even though I would have wanted to be able to do that*. A typical scenario found in the data was that a second-line manager was the head of a top-management team comprising unit heads of different units, for example, healthcare centres or wards. In this top-management team, there were representatives from the administration, for example, a development leader, an HR representative and a controller. However, these representatives were not managers with formal mandates; they delivered information that someone else has decided. The second-line manager was not their manager and was, at the same time, one level above them in the overall organisational hierarchy. One second-line manager explains: *They are not my employees, but I need to make sure that they are treated fairly … . I need to step in and align the different perspectives.*

Some managers spoke about loyalty, and how there was a sense of distrust between the line organisation and ‘the administration’. The issue of loyalty was described as an invisible *‘feeling that was always present’,* affecting the managers. One second-line manager in the line-organisation discusses the issues of loyalties that she experienced was always there yet not spoken about: … *there is this … subtle thing that you may not really talk about that is constantly present and influencing. Where does the loyalty lie? What can I talk about? Can trust these people [belonging to the administrative unit]. Can I exchange some strategic talk here now so that we can get a good budget for next year, or will that lead to you giving me a fine?* This relates to the issue of who your colleagues are. Are you employed in the same organisation or in a different organisation? The manager continues:*… they* [the administrative people*] were not my employees.* Not being one’s employee also meant having different targets that sometimes were contradictory, and, in those cases, the one who sets the salary is who you are loyal to. The manager ends by saying: *So, they were not as close to me … when you talk about that loyalty, it’s also about who sets my salary.*

#### Taking ‘sides’: first-line managers for the core operations distinguish themselves from others

Some managers described how they actively took sides in the ‘conflict’ between the administration and the core operations, the conflict being defined as who should make decisions on clinical work and practice. By joining in the jargon of their employees, consciously deciding to wear scrubs and ‘be one in the gang’, first-line managers would mark that they were on ‘the right’ side. Some clinical managers used the conflict between clinicians and administrative units to create buy-in from their staff, joining the jargon. This was, for example, shown in the following quote by a first-line manager: There are times when I take-sides and join my staff, and I say thing like: *‘They have no idea over there at the headquarters … about us and our reality’.* Sometimes, this was done not because the managers entirely believed it, but because it would strengthen their credibility amongst their personnel.

First-line managers’ stories revealed a deep personal investment in their staff, often describing themselves as taking on a near-parental role, for example, assisting employees with personal matters such as finding housing. They often described *trying to patch a broken system*, working long hours and remaining constantly available to step in when needed. At the same time, they expressed a sense of imbalance, feeling that their own managers and higher-level leaders did not offer them the same level of support. To signal investment and engagement, wearing scrubs was important for first-line managers. A first-line manager describes the reason for her to wear scrubs: *It’s a signal that you’re ready, that you’re not sitting on a higher chair or anything like that, but you are ready to dig in.* In working hard and being engaged with their staff on a personal matter, this was contrasted with the lack of commitment from second-line managers, who were seen as less engaged and not dressed to work at a hospital. From the data gathered for this study, they were also less engaged in operative issues, in the sense that they did not personally seem to invest in their staff in the same way; they did not wear scrubs and did not engage in the clinical work. The first-line managers’ stories were that they were doing much, whereas others were doing too little: *There’s no one who works as hard as first-line managers and who cares as much about their patients. And then, of course, you want there to be the same level of commitment in healthcare administration, throughout all levels, and not this kind of laid-back attitude.* (Third-line manager).

#### Who works for whom?

While administrative managers described not being taken seriously, clinical line managers expressed frustration over a perceived lack of support. They questioned why their organisations should have to adjust to administrative structures, especially given that they were the ones financing support functions. Line managers felt that the administration appeared more focused on improving its own processes rather than supporting clinical work. One line manager describes a particularly frustrating example: *they have introduced “phone hours”. i.e., time-slots when we can reach them. When you have questions about for example our time management program. That is one of those incredibly strange moves in my eyes and all our eyes. We finance that activity with our revenues. They are a support function for us! Then we need to adjust to their phone hours instead of meeting patients.*

### Conflicting views on where power resides and who has it

#### Administration: supporting or steering?

A common responsibility for administrative staff was to implement various tools and measures. However, a recurring view among line organisation managers was that the administration lacked an understanding of the realities of clinical work, and that the solutions being implemented did not align with operational needs. Tensions often arose when the administration was tasked with introducing changes that did not fit the existing structure of healthcare delivery. While the core operations sought to maintain established practices, ‘the administration’ aimed to reform them. One example of this disconnect was the issue of digitalisation. According to administrative units, digitalisation was intended to support a more patient-centred approach and enable co-creation of services, allowing patients to choose how they access care. However, the core operations staff argued that the way digitalisation was implemented did not align with their organisational structure, and they were unwilling to adapt to these changes. For example, when discussing new digital solutions to get in contact with care premises, a common perception was this: *It is one thing that a person who knows IT can manage these things … but when [generic name of old lady] aged 85 is to connect to a different system. It does not work … then our/the care perspective becomes to explain that this is not how we work … your [reffering to organisational developer] ideas will not work and will not be used* (Second-line manager, line organisation).

This was related to issues of mandates. Administrative managers expressed frustration as they were tasked to implement digital solutions to ease patient participation, spent a considerable amount of time on developing tools and measures to enable this, only to discover that first-line managers could just decide not to implement these tools and measures. A manager for an administrative unit, tasked to coordinate the care processes for a particular disease, explains: *[name of other organisation] has developed [name of digital solution], which is about how the patient’s path through the hospital should look like … then it has turned out that this is implemented differently. Some wards have implemented it, and some haven’t because they think that ‘no, but we shouldn’t have to do that’, right? We say: “It’s not a discussion, like, it should just be implemented!” But I have no mandate at all, really. … It is up to the managers of the clinics and wards, and they do not want to implement it.*

Another manager at an administrative unit complains that the clinical first-line managers have not managed to inform their personnel about current troubles that the administration is now working on to fix: *I have come to units where the doctors don’t even know that we have poor telephone access. ‘What do we have that?’ And of course, if you don’t know that it’s a problem, then why would they welcome us?*

This echoes the feeling from the administrative manager who expressed: *We could work as much as possible, and still everyone is disappointed because they think we’re supposed to be doing something else.*

Notably, the line managers reflected upon the fact that they, in general, did not know much about the administrative side of healthcare. One third-line manager reflected: *I did not even know there was a financial organisation before I got my first paycheck.*

#### Power is always somewhere else

In relation to where power resides, healthcare was described as a system, driven by a kind of ‘force’ that no one had the ability (or power) to control, which made it hard to collaborate. Members high up in the hierarchy, including representatives of top management, labelled the healthcare organisation ‘the system’ and described it like this: ‘*The system’ is not designed for us to be able to show understanding for each other.* First-line managers generally expressed that they were controlled by ‘upper management’, or more vaguely ‘those up there’ and would typically not mention their own power and possibility to influence organisational matters, but rather how they were exhausted due to delivering everything that ‘those up there’ had told them to do. One first-line manager described this as ‘being stuck in a tumble dryer’ with all demands making everything spin. However, on the contrary, representatives of the highest management typically alluded to the fact that first-line managers and clinical personnel are those with power: *Both the formal power lies in the line, as well as the informal power, that is, what can be decided regarding resources. Each doctor has an enormous amount of decision-making power regarding the type of treatment, which medications, which care times, and how resources should be utilised. It is delegated all the way out (Director).*

In addition to alluding power to the front-line staff, the higher up you came in the organisation, the power was either attributed to the very ‘bottom’ or the politicians. The politicians were, on the one hand, blamed for bad decisions, but there was also frustration from some administrative units that they had been privileged by the politicians and that this was not understood by the line organisation. One manager for an administrative unit describes how members of the clinical line organisation question him and how his organisation can have so much money: *[they say] you get so much money! [Then I say] OK, but if you have a problem with that, maybe you should go into politics and not go at us who work to implement the political decisions!* He and others referred to that if core operations want to change how things are done, they need to vote for other politicians, but often the core operations would not know of the power that politicians have over healthcare organising. Here, in this case, tensions could be argued to arise because different parts of the system did not know how healthcare is governed. Thus, tensions are then a consequence of a lack of knowledge of how the formal power is organised, which leads back to the idea of how to manage and ease conflicts and tensions: through education.

## Discussion: healthcare managers, conflicts, tensions and acting as reflexive public professionals with a holistic viewpoint

The focus on the increasing tensions and conflicts in this paper, between the administration and the core operations against the backdrop of increasingly complex institutional contexts, and the increasing administrative layer without steering (cf. [8, 29]) reveals how public healthcare managers need to integrate different professional groups and different modes of governance. As one example, the administration tried to implement measures that came as a response to new public governance ideals, such as increasing patients’ involvement in their care through digital solutions, with new public management measures of standardisation that the core operations despised (cf. [16]), leading to tensions and conflicts. This highlights the need to understand the co-existence of various ideals in practice for healthcare managers and skills in how to manage these.

This paper addressed the following research questions: How do healthcare managers, both those representing ‘the line organisations’ and those representing ‘the administration’ navigate tensions and conflicts between them, and how does this affect the possibility of acting like public professionals?

First, for line organisation managers, particularly for first-line managers, integrating ‘the administration’ with ‘the core’ seems difficult due to a lack of knowledge, as well as a lack of willingness and interest, and identification with the overarching system in which the different groups exist. For the administrative managers, integration and managing tensions were hard due to a lack of legitimacy, as they belonged to a sub-system (i.e. administration) that was not seen as equally valuable as other sub-systems, particularly the core operations [3, 19, 20, 22]. This lower status was visible concerning the issue of competence. A lack of steering of the growing administration was, for example, manifested in conflicting views on what competence was needed to work with such things as finances and fire safety. Here, it became obvious that administrative work was not seen as equally ‘real’ and important as clinical, operative work, and competence needed was much more up to individual managers’ liking than to an overarching agenda. In addition, the data showed how the administration managers were not given proper mandates and how the clinical-line managers (lower or at an equal level in the formal hierarchical order) were still the ones deciding, for example, whether to implement digital solutions. Thus, while the overarching organisation responded to coordination and development by introducing roles that were to facilitate collaboration between administrative units and develop new solutions, it was still line managers at lower levels who made the decisions in practice to implement new solutions or not. This highlights the need to understand healthcare as a hierarchy in which the different organisational units do not align in practice, and the ‘core operations’ are still powerful, despite efforts to challenge this [3].

That power is always somewhere else was a perception by the interviewed managers, but it is also a structural feature of how healthcare is organised. In healthcare organisations power is inherently fragmented. In that sense, the managers were right; some aspects of power are always somewhere else. Nevertheless, while power is in part always somewhere else, it could be argued that public healthcare managers ought to recognise how their perceptions and actions contribute to consolidating power in certain places in the organisation. Their tendency to use ‘sweeping’ expressions could also be a way of not claiming responsibility for their own power. Overall, when discussing solutions to tensions, organisational solutions tended to be vague and abstract. When talking about how to meet and integrate, it was often very ‘distant’ wording, cliches and platitudes, such as ‘there should be a will to bridge the different functions’, ‘we would like more collaboration’, ‘the administration needs to “keep an ear on the rail”’. Hence, integration appeared to be wanted on an abstract level but did not result in concrete integrative practices (cf. [11]).

The data showed that some line managers (typically those with a physician background) describe a ‘transition’ once they moved up in the hierarchy, a transition during which they began to understand how the healthcare system works and the meaning and value of ‘the administration’. In one way, this implies a willingness from line organisation managers to learn about the system, but the question remains whether this understanding leads to a desire to employ a ‘holistic viewpoint’ or not.

The solutions to the tensions and conflicts that the managers proposed did not consider more overarching questions related to the purpose of the organisations, nor the purpose of the administration as such (rather than the sweeping idea that they should support the core operations). This relates to the proposed need for strategic leadership for public healthcare managers and the need for public professionals to find common meanings that transcend individual professional groups and units, as suggested by Hendrikx et al. [1] (See also, for example [36], regarding managers and delivering on purpose).

Second, ‘Taking sides’ with their personnel as the first-line managers were shown to do, could be interpreted both as a strategic choice and/or a coping mechanism. Both interpretations, however, imply that managers might need to rethink why they employ either of these strategies. Furthermore, this finding highlights the role complexity for managers, as, on the one hand, aligning with staff can be considered a demonstration of empathy and trustworthiness, in line with the basic attributes needed for public professionals identified by Hendrikx et al. [1]. However, simultaneously, it probably does not increase ‘other partners’ collaborative capacity’, also a skill needed for public professionals, exemplifying the need for healthcare managers to balance various demands in practice. The tendency for line managers to align with their professional group and delegate integrative tasks reflects broader patterns that could be interpreted as both conflict avoidance and thereby contributing to increased tensions. As Rossi and Tuurnas [2] suggest, such avoidance may hinder the holism that public professionals are supposed to facilitate.

According to Hendrikx et al. [1], reflexive public professionals should be able to ‘bridge worlds’, facilitate collaboration and engage in ‘big picture’ thinking. However, the findings from this study show how managers, particularly line organisation managers (first-line), took sides and felt they needed to ‘go with their personnel’ to get their buy-in. Thus, rather than bridging worlds, they were engaged in endeavours that could be said to keep ‘worlds’ apart. In addition, they described how they put issues of collaboration and navigating organisational structures onto organisational developers, who often would belong to other sub-systems and not have proper mandates. This reflects uncertainty about their role in a fragmented system such as healthcare (cf. [22], which means that the managers did not engage in the skills that Hendrikx et al. [1] propose are essential for public professionals in today’s complex institutional environments, such as healthcare. The findings thereby reflect a gap in strategic leadership capacity and a lack of perceived legitimacy to act across subsystems [1] for healthcare managers.

The findings in this paper open for discussion regarding the future role of public healthcare managers. A suggestion for future research is to engage public healthcare managers in discussions on how their ways of navigating tensions and conflicts relate to the skills that public professionals are argued to need. This research ought to study the shift in how we conceptualise healthcare managers: from unit-bound leaders to public professionals who mediate across fragmented systems. Future research needs to explore how to equip managers with the skills and support needed to fulfil this role and discuss the role of public healthcare managers in intersectional modes of governance.

## Conclusions

Tensions between ‘the line organisation’ and ‘the administration’ have of late increased in Swedish healthcare, but the role of managers in and for these tensions has not been investigated. By exploring how Swedish public healthcare managers, both from ‘the administration’ and the line organisation, understand and navigate tensions and conflicts between them, this paper contributes to an understanding of the challenges facing healthcare managers in today’s public healthcare organisations, characterised by increasingly complex institutional contexts.

For healthcare managers to be able to be reflexive public professionals and employ a ‘holistic viewpoint’, it is important to increase understanding of how they deal with and experience current conflicts and tensions. By focusing on tensions and conflict, this study enabled an increased understanding of what ways healthcare managers are able, or not, to act as public professionals with a holistic viewpoint, where tensions and conflicts are reinforced and where managers can start making sense of their experiences if conflicts and tensions are to be generative. Healthcare managers struggle to act as public professionals in a fragmented system marked by conflicting logics and unclear mandates, and addressing these challenges requires rethinking managerial roles and their prerequisites to foster strategic, integrative leadership across organisational boundaries. Today, rather than bridging divides, public healthcare managers reinforce these through alignment with their professional groups.

There seems to be a gap between experiencing tensions and conflicts and reflecting on how to solve them, indicating a need for forums in which these things can be discussed. Recognising and working through conflict is an opportunity for growth but requires public healthcare managers to look beyond their organisations and professions, to employ the holistic viewpoint. This is something for future studies to focus on: how to best help healthcare managers make sense of their experiences, and to make the tensions and conflicts they encounter generative.

In this paper, the focus was on the administrative units and their actors, and how to integrate these with the line organisation (and vice versa). For future studies, it would be good to keep the issue of ‘administration’ more open, since administrative tasks are also part of the task of managers at all levels, and perhaps increasingly so, as illustrated by the consequences of digitalisation. The low status of administration in the healthcare system and how it is distinguished from (clinical) management is indeed a very interesting issue that needs further exploration.

This study was set in public healthcare organisations in Sweden. In future studies, focusing on the relationship between line organisation and ‘the administration’ in healthcare and the role of managers, there is merit in more thoroughly tending to the institutional arrangements and policies in Sweden and how they affect practice, as well as looking into other countries and their institutional arrangements.

## Electronic supplementary material

Below is the link to the electronic supplementary material.

Supplementary Material 1

## Data Availability

The data collected and analysed in this manuscript are not publicly available due to participants did not consent to public availability of data. Aggregated data in Swedish are available from the corresponding author on reasonable request.

## References

[1] Hendrikx W, Kuiper M, van Gestel N. Engaging professionals in the strategic renewal of public services: a literature review and research agenda. Public Manag Rev. 2022.

[2] Rossi P, Tuurnas S. Conflicts fostering understanding of value co-creation and service systems transformation in complex public service systems. Public Manag Rev. 2019;23(2):254–75. 10.1080/14719037.2019.1679231.

[3] Kirkpatrick I, Zardini A, Veronesi G. Management reforms, re-stratification and the adaptation of professional status hierarchies: the case of medicine in publicly owned hospitals. Public Manag Rev. 2023.

[4] Alamaa L, Hall P, Löfgren K. Why are organisational professionals expanding in the Swedish public sector? The role of accountability. Public Policy Adm. 2025.

[5] Glouberman S, Mintzberg H. Managing the care of health and the cure of disease-part I: differentiation. Healthcare Manag Rev. 2001 Winter;26(1):56–69. Discussion 87-9. 10.1097/00004010-200101000-00006. PMID: 11233354. 10.1097/00004010-200101000-00006 11233354

[6] Kanon M, Andersson T. Working on connective professionalism: what cross-sector strategists in Swedish public organizations do to develop connectivity in addressing ‘wicked’ policy problems. J Retailing Professions Organ. 2023;10(1):50–64.

[7] Kirkpatrick I, Altanlar A, Veronesi G. Corporatisation and the emergence of (under-managered) managed organisations: the case of English public hospitals. Organ Stud. 2017;38(12):1687–708. 10.1177/0170840617693273.

[8] Alvehus J, Kastenberg Weichselberger G. Administration i offentlig sektor - Om expansion, elevering och mittokrati. 2024.

[9] Svensson I, Andersson T. How perceptions of time and place construct two stories concerning status and privilege for clinicians and administrators in healthcare organizations. J Prof Organ. 2025. Forthcoming.

[10] Haring M, Freigang F, Amelung V, et al. What can healthcare systems learn from looking at tensions in innovation processes? A systematic literature review. BMC Health Serv Res. 2022;22:1299. 10.1186/s12913-022-08626-7. 10.1186/s12913-022-08626-7 PMC9617372 36307839

[11] Keller EJ, Chrisman HB, Collins JD. The growing pains of physician-administration relationships in an academic medical centre and the effects on physician engagement. PLoS One. 2019;14(2). 10.1371/journal.pone.0212014 PMC6373942 30759151

[12] Martin GP, Currie G, Finn R. Reconfiguring or reproducing intra-professional boundaries? Specialist expertise, generalist knowledge and the ‘modernization’ of the medical workforce. Soc Sci Med. 2015;126:117–24. 10.1016/j.socscimed.2014.12.007. 10.1016/j.socscimed.2009.01.006 19201073

[13] Denis JL, van Gestel N. Medical doctors in healthcare leadership: theoretical and practical challenges. BMC Health Serv Res. 2016;16(Suppl 2):158. 10.1186/s12913-016-1392-8. 10.1186/s12913-016-1392-8 PMC4896273 27230551

[14] McGivern G, et al. Hybrid manager-professionals’ identity work: the maintenance and hybridization of medical professionalism in managerial contexts. Public Admin. 2015;93(2):412–32.32.

[15] Noordegraaf M. Hybrid professionalism and beyond: (New) forms of public professionalism in changing organizational and societal contexts. J Prof Organ. 2015;2(2):187–206.

[16] Eriksson E, Andersson T. The ‘service turn’ in a new public management context: a street-level bureaucrat perspective. Public Manag Rev. 2023;26(7):2014–38. 10.1080/14719037.2023.2241051.

[17] Currie G, White L. Inter-professional barriers and knowledge brokering in an organizational context: the case of healthcare. Organ Stud. 2012;33(10):1333–61. 10.1177/0170840612457617.

[18] Cregård A, Forsberg T, Berntsson E. (reds) Samspel i kommunal administration: Lagspel, dragkamp eller hierarki? Lund: Studentlitteratur; 2023.

[19] Kuhlmann E, Rangnitt Y, von Knorring M. Medicine and management: looking inside the box of changing hospital governance. BMC Health Serv Res. 2016;16:160. 10.1186/s12913-016-1393-7. 10.1186/s12913-016-1393-7 PMC4896265 27230654

[20] Sørensen et al. Leading nurses in dire straits: head nurses’ navigation between nursing and leadership roles. J Nurs Manag. 2011 May;19(4):421–30. 10.1111/j.1365-2834.2011.01212.x. Epub 2011 Mar 29. PMID: 21569139. 10.1111/j.1365-2834.2011.01212.x 21569139

[21] Kislov R, Waterman H, Harvey G, et al. Rethinking capacity building for knowledge mobilisation: developing multilevel capabilities in healthcare organisations. Implementation Sci. 2014;9:166. 10.1186/s13012-014-0166-0. 10.1186/s13012-014-0166-0 PMC4234886 25398428

[22] Vik E, Hansson L. Contingency and paradoxes in management practices-development plan as a case. J Health Organ Manag. 2024;38(9):72–88. 10.1108/JHOM-06-2022-0175. 10.1108/JHOM-06-2022-0175 PMC10986774 38448231

[23] von Knorring M, Alexanderson K, Eliasson MA. The manager role in medical management. Eur J Criminol Public Health. 2014;24(Suppl 2):163064. 10.1093/eurpub/cku163.064.

[24] Freidson E. Professionalism, the third logic: on the practice of knowledge. Chicago (IL): University of Chicago Press; 2001.

[25] Waring J. Restratification, hybridity and professional elites: questions of power, identity and relational contingency at the points of ‘professional-organisational intersection’. Sociol Compass. 2014;8(7):688–704. 10.1111/soc4.12178.

[26] Evetts J. New professionalism? Challenges and opportunities. Curr Sociol. 2011;59(4):406–22. 10.1177/0011392111402585.

[27] Fjällström P. Standardized cancer patient pathways: a perspective from primary care in northern Sweden. [doctoral thesis]. Umeå: Umeå University; 2024.

[28] Kreindler SA, Larson BK, Wu FM, Gbemudu JN, Carluzzo KL, Struthers A, Van Citters AD, Shortell SM, Nelson EC, Fisher ES. The rules of engagement: physician engagement strategies in intergroup contexts. J Health Organ Manag. 2014;28(1):41–61. 10.1108/JHOM-02-2013-0024. PMID: 24783665. 10.1108/JHOM-02-2013-0024 24783665

[29] Hall P. Varför ökar den offentliga byråkratin i Sverige? Malmö University Press; (2025). 10.60156/kriterium.65.

[30] Vik E, Hjelseth A. Integrasjon av helsetjenester: åtte teser om samhandling i en funksjonelt differensiert helsetjeneste. Tidsskr Samfunnsforsk. 2022;63(2):122–40. https://www.idunn.no/doi/pdf/10.18261/tfs.63.2.3.

[31] Fox S, Comeau-Vallée M. The negotiation of sharing leadership in the context of professional hierarchy: interactions on interprofessional teams. Leadership. 2020;16(5):568–91. 10.1177/1742715020917817.

[32] Høiland GCL, Klemsdal L. Organizing professional work and services through institutional complexity - how institutional logics and differences in organizational roles matter. Hum Relat. 2022;75(2):240–72. 10.1177/0018726720970274.

[33] Desmidt S, Heene A. Mission statement perception: are we all on the same wavelength? A case study in a Flemish hospital. Healthcare Manag Rev. 2007;32(1):77–87. 10.1097/00004010-200701000-00010 17245205

[34] Braun V, Clarke V. Using thematic analysis in psychology. Qual Res Psychol. 2006;3(2):77–101. 10.1191/1478088706qp063oa.

[35] Gioia DA, Corley KG, Hamilton AL. Seeking qualitative rigor in inductive research: notes on the Gioia methodology. Organ Res Methods. 2013;16(1):15–31. 10.1177/1094428112452151.

[36] By RT. Leadership: In pursuit of purpose. J Educ Chang Change Manag. 2021;21(1):30–44. 10.1080/14697017.2021.1861698.

