# Supplementary material for: Taking sides or bridging worlds? Managerial responses to conflicts and tensions between the core operations and the administration in healthcare
Source: BMC Health Serv Res. 2025 Nov 8;25:1453. doi: 10.1186/s12913-025-13659-9 (PMC12595710; doi:10.1186/s12913-025-13659-9)
Supplement: Supplementary file 1 — Supplementary Material 1 [file 12913_2025_13659_MOESM1_ESM.pdf]

Interview guide with topics and examples of questions

All interviews started with a background to the project and the researchers' identification of tensions between occupational groups based on both general and scientific media outlets, Linked in posts, etc.

- The first topic concerned the interviewee's position in his/her organisation. Questions such as: Could you describe your role? Who do you report to? What type of professionals are on your team? What do you do in your work? What is your background/prior experience?

- The second topic concerned current tensions in health care organisations, between administrative and clinical personnel. Questions such as: Do you recognise these tensions? Can you give examples of how you have encountered them? What is your role in relation to these tensions? Could you give concrete examples of when you have noted these tensions, what did you do? What did others do?

- What do you see as a solution to current tensions in healthcare organisations?

Questions could vary depending on the interviewee and the individual interview.
